# Future prospects for human genetics and genomics in drug discovery

**DOI:** 10.1016/j.sbi.2023.102568

**Published:** 2023-03-22

**Authors:** Maya Ghoussaini, Matthew R. Nelson, Ian Dunham

**Affiliations:** 1 Wellcome Sanger Institute, Wellcome Genome Campus, United Kingdom; 2 Open Targets, Wellcome Genome Campus, United Kingdom; 3 Deerfield Management Company, L.P., New York, NY; 4 European Molecular Biology Laboratory, European Bioinformatics Institute (EMBL-EBI); Wellcome Genome Campus, United Kingdom

**Keywords:** Genetics, Genomics, Drug Discovery, GWAS, Sequencing

## Abstract

Evidence from human genetics supporting the therapeutic hypothesis increases the likelihood that a drug will succeed in clinical trials. Rare and common disease genetics yield a wide array of alleles with a range of effect sizes that can proxy for the effect of a drug in disease. Recent advances in large scale population collections and whole genome sequencing approaches have provided a rich resource of human genetic evidence to support drug target selection. As the range of phenotypes profiled increases and ever more alleles are discovered across worldwide populations, these approaches will increasingly influence multiple stages across the lifespan of a drug discovery programme.

## Introduction

Over the past 75 years, the development of new therapeutic drugs for serious illness has had a major impact on improving lifespan and quality of life. Early successes were mostly characterised by chance observations and application of emerging chemistry on successful folk medicines [1]. Over time, drug discovery has evolved to be a targeted endeavour, with mostly protein targets selected based on growing understanding of disease biology with cellular and animal models providing most of the insights to support clinical translation. Unfortunately, this period has also seen research and development (R&D) productivity decline dramatically. In the 1970’s, about 10 new drugs were approved for every billion US dollars R&D spent. By 2000, the same investment yielded less than one approved drug per year [2]. New technologies increased the scale and cost of R&D capabilities, but low success rates in clinical development largely driven by poor effects on disease outcomes resulted in ever lower productivity. However, over the past decade we have seen evidence that this decline may be stabilising, and perhaps even reversing [2].

These productivity gains are increasingly being driven by improvements in understanding of the causes of rare disease, cancer and, more recently, common complex disease provided by genetics and genomics. Identifying genes underlying rare genetic disease has opened many of them to effective therapeutic development, with much higher average development success rates than seen in most other clinical areas [3]. Diagnosis of cancer at the causal genetic and genomic level has resolved apparently similar diseases at the histological scale to often a highly heterogeneous set of diseases from the perspective of the underlying mutations. Designing therapies with these differences in mind, and including them in patient screening, improves development success rates at least two fold [4]. In addition to these “simple” conditions, at least from a causal genetics perspective, what has been equally remarkable is that evidence from genes with subtle effects on the risk of more complex, multifactorial diseases, result in drug mechanisms that are at least twice as likely to succeed in clinical development compared to drugs lacking such support. Insights into these more marginal causal genetic factors can be leveraged to identify and prioritise more effective drug discovery opportunities [5][6]. Moreover, a recent retrospective analysis found that 2 out of 3 of the 2021 US Food and Drug Administration (FDA)-approved drugs are supported by human genetics evidence [7]. Taken together, integration of genetic evidence into drug discovery can dramatically improve overall R&D productivity.

### Rare Disease Genetics

**R**are genetic diseases are usually attributed to protein-coding mutations affecting a single gene (monogenic). Their pathological mechanisms tend to be better understood facilitating translation of these insights into drug development. Examples include mutations in the CFTR gene in cystic fibrosis, in the beta-globin gene in sickle cell anemia, and in Huntington gene HTT in Huntington’s disease. Advances in technology including access to whole exome and genome sequencing have revolutionised our understanding of the genetic basis of a broad spectrum of rare diseases and interpretation of their clinical consequences [8].

Often, knowledge of the underlying causal gene in rare disease can be directly utilised for drug development. Duchenne muscular dystrophy (DMD) is a lethal disease caused by deletions in one or more exons of the dystrophin gene (mainly exons 45, 48 and 51) causing a reading frameshift and premature termination, resulting in a truncated dysfunctional protein and consequently muscle degeneration [9]. This understanding has enabled development of targeted exon skipping therapies. Casimersen is an antisense oligonucleotide drug designed to bind to exon 45 of the dystrophin pre-mRNA, leading to exon removal during splicing, thereby correcting the reading frame to give a shorter but functional dystrophin protein in treated patients [10]. Casimersen received its first approval In 2021 [11].

**Figure 1:**
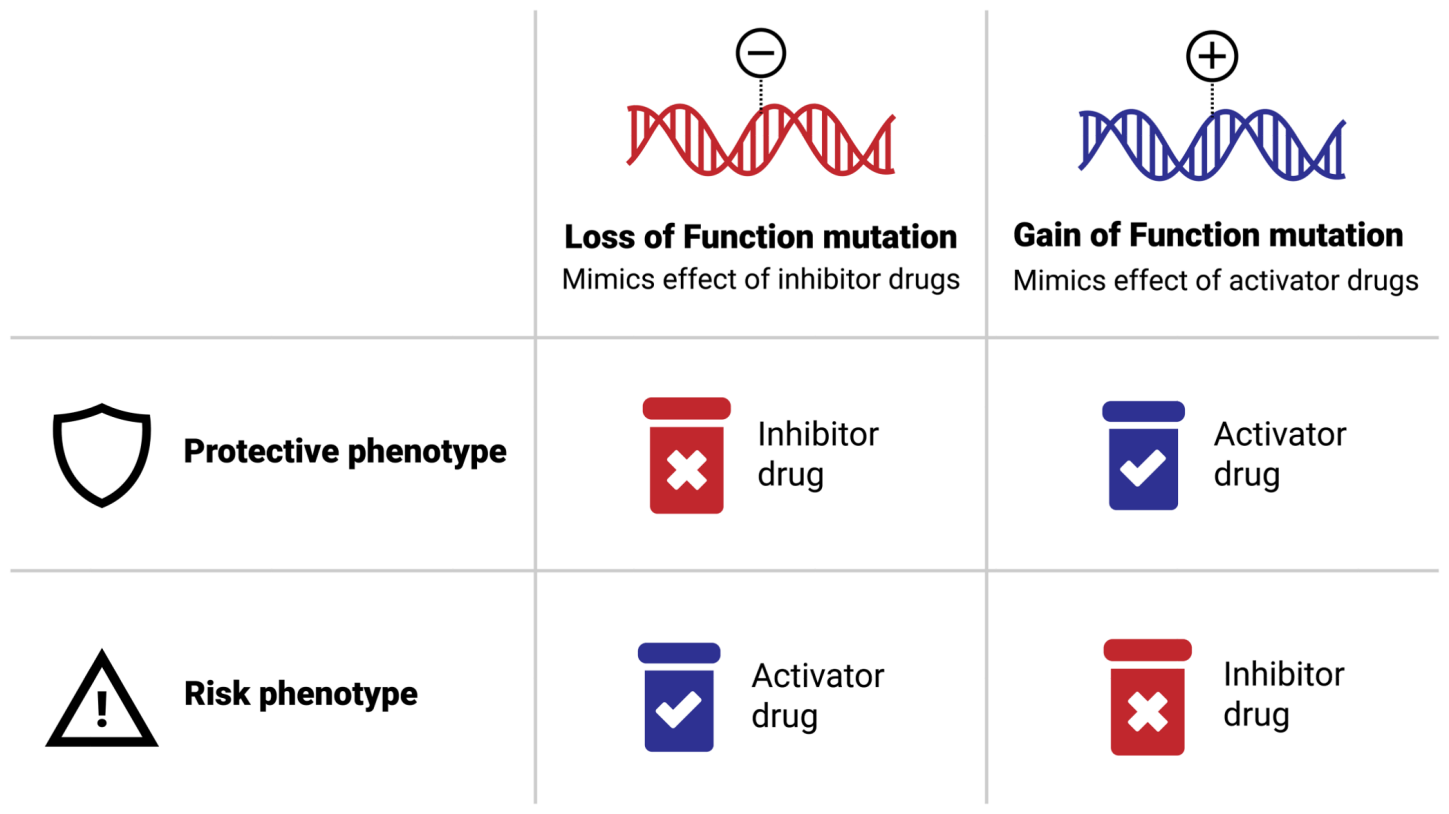
Framework used to derive the desired therapeutic target modulation in the context of a phenotype. Loss of function mutations associated with protective phenotypes or gain of function mutations associated with risk phenotypes make the gene an attractive target for inhibitor drugs. Conversely loss of function mutations associated with risk phenotypes or gain of function mutations associated with protective phenotypes favour drugs with an activator mechanism of action.

Rare disease genetics can also inform drug discovery in common disease. Identification of a spectrum of rare protein-coding mutations within a gene ranging from loss of function (LoF) to gain of function (GoF) mutations combined with phenotypic and clinical information can inform the potential efficacy and toxicity from therapeutically modulating that protein in humans [12]. Given that the majority of drugs are inhibitors, attention has turned towards rare LoF mutations that protect against disease and gain of function mutations that increase disease risk (Figure 1). A well known example is the proprotein convertase PCSK9. LoF mutations in PCSK9 reduce serum LDL cholesterol levels and protect against coronary heart disease while GoF mutations increase risk of hyperlipidemia, a condition that significantly increases early-onset cardiovascular disease risk [13]. Functional experiments demonstrated that, after entering circulation, PCSK9 binds LDL receptors promoting their endocytosis and degradation and reducing cholesterol removal from blood [14]. These evidence combined led to development of PCSK9 inhibitors and by 2017 two (alirocumab and evolocumab) were approved by the FDA to treat patients with high cholesterol unresponsive to statins or diet [15] [16] [17].

### Common Disease Genetics

Many complex diseases including neurodegeneration, auto-immunity and type 2 diabetes are partially driven by many common genetic variants, each having a modest individual effect on the phenotype [18]. Genome-wide association studies (GWAS) approaches were designed to map this polygenic architecture by scanning thousands or millions of common variants in the genome for the subset present at significantly higher frequency in patients compared to controls. Unlike rare disease-causing mutations, most GWAS-associated variants fall in the non-coding part of the genome (mainly intergenic and introns) suggesting that they affect gene expression rather than protein structure. The connection of associated variants with their likely targeted gene is laborious requiring integration of a range of transcriptomic, proteomic and epigenomic data across different cell types and tissues [19]. Functional validation is often conducted to pinpoint the likely causal variant and the actual causal gene. Other statistical approaches, such as Mendelian randomisation [20][21], can be used post-GWAS to implicate a protein or a gene causally with an outcome [22].

The first GWAS for any common human disease was in 2005 for age-related macular degeneration (AMD), a condition with complex etiology including gradual loss of central vision [23]. Two decades prior, genetic linkage analysis in families implicated a region of chromosome 1 in the disease [24]. That same region was identified in the GWAS and a common coding missense variant in the complement factor H gene (CFH) was strongly associated with increased AMD risk [23]. Over the next decade, additional common variants were identified in or near other complement genes including CFB, C2, C3, C9 and CFI further implicating the complement pathway in AMD [25]. Complement inhibitors are either currently under development or being repurposed to treat AMD [26].

### Allelic series

Recent analysis of rich genotypic and phenotypic datasets suggests the historical separation of monogenic and polygenic diseases may be too simplistic. In reality most genetic diseases and complex traits have risk alleles distributed across a continuous spectrum of frequencies and effect sizes [27] [28].

**Figure 2:**
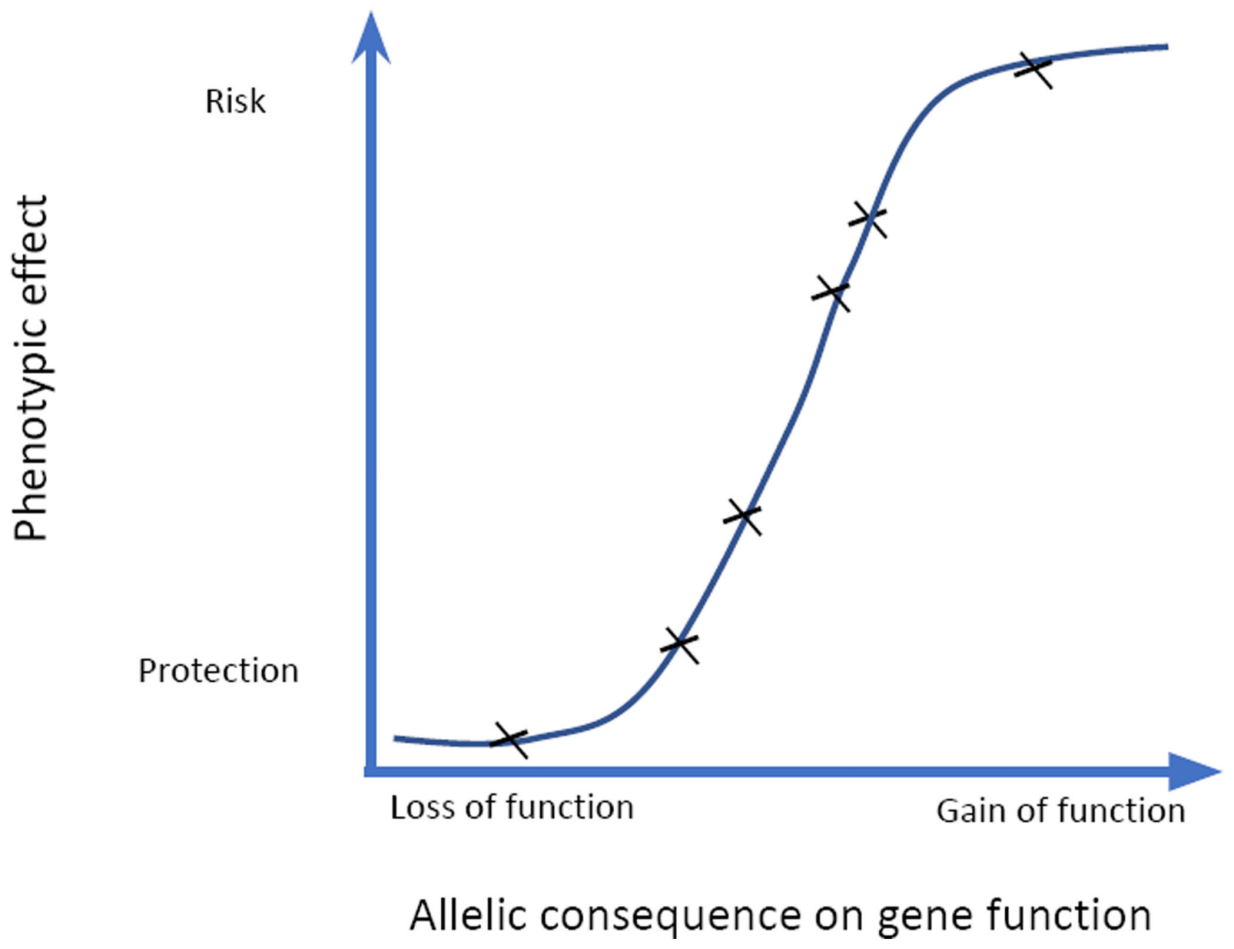
Schematic dose-response curve. A range of alleles perturbing target function is used to estimate the impact on clinically-relevant phenotypes. In this case LoF alleles have a protective effect on disease risk as for PCSK9 where LoF reduces serum LDL cholesterol levels and protects against coronary heart disease. For the more common case where LoF increases disease risk the y axis would be reversed.

An allelic series refers to a set of naturally occurring rare and common variations within the same gene with different effects on phenotype severity. This can help estimate the quantitative relationship between gene function and the clinical phenotype generating a dose-response curve approximating that of a potential drug [12] (Figure 2). For instance, loss of function mutations identified through exome/whole genome sequencing could proxy for the effect of complete inhibition of a gene product, while milder common regulatory variants downregulating the gene identified through GWAS could be equivalent to partial inhibition.

By combining rare and common variant signals from whole exome sequencing (WES) and GWAS data in half a million UK Biobank participants, Regeneron scientists recently identified that rare variant associations were often found near a GWAS signal for the same trait and the majority remained significant after conditioning on the common signal [29] suggesting that many novel allelic series could be found. Leucine-rich repeat kinase 2 (LRRK2) is a serine/threonine-protein kinase that phosphorylates a range of proteins and affects neuronal plasticity and autophagy. Rare GoF mutations in LRRK2 with high and moderate penetrance and common regulatory variants affecting LRRK2 gene expression significantly increase Parkinson’s disease risk [30], suggesting that inhibiting LRRK2 kinase activity may be therapeutic. Substantial pre-clinical and clinical effort has been undertaken to reduce the toxicity linked to LRRK2 hyperactivity yielding several inhibitor drugs currently in clinical trials [31].

### Future Prospects

Growing evidence for genetic support as a positive prognostic indicator for clinical drug trials and increased investment of pharmaceutical companies in genetics (and geneticists) [32] suggests that the value of genetic evidence will continue to grow. Increasingly, evidence will come from systematic analysis in populations such as UK Biobank [33], Finngen [34] or 23andMe [35] and other national biobanks with increased use of whole exome and genome sequencing integrated with electronic health records (EHRs). Other notable initiatives among many include the NIH’s AllofUs programme, the Danish Genome project, the Million Veterans Program and expanded genome sequencing in the UK’s National Health Service. With larger populations and broad phenotyping, ever smaller but significant effects on relevant traits in disease will be revealed, as well as drug repurposing opportunities or potential safety liabilities from genetic analysis across traits (PheWAS) [36]. Furthermore with increased population diversity, other population-specific effects will be detected absent from the primarily European populations studied so far. Differences in linkage disequilibrium structure between populations can also help to resolve the causal gene [37]. It will become possible to generate gene function-clinical phenotype combinations for nearly every gene in the genome. In fact, sequencing ~5 million individuals is predicted to identify more than 500 heterozygous LoF carriers for around 15,000 genes [29]. Furthermore, accessing consanguineous populations where homozygous LoF are prevalent with recall by genotype for deep carrier phenotyping will speed up our understanding of gene function-clinical phenotype relationships [38,39]. Each of these associations is a potential therapeutic hypothesis to test. As this map of the genetic burden in disease improves, drug discovery scientists will also look to network analysis to identify suitably tractable targets connected to but not directly implicated by genetic evidence [40].

The increased number of hypotheses to test will require higher throughput approaches to target validation. Genetics and genomics approaches can assist here with increasingly sophisticated techniques including high throughput gene editing and single cell analysis to unpick the relevant biological mechanisms. Initiatives such as the Human Cell Atlas [41] and the Atlas of Variant Effects [42] are just some of the resources providing this information.

We anticipate continued advancements in machine learning technology, particularly applications of deep neural networks, to have an increasing impact on our ability to integrate vast, multi-modal genetic and genomic data to better understand how genes and the molecular pathways they encode affect disease [43]. Just as AlphaFold [44] sparked a variety of entirely new structural biology research applications, new approaches to predicting and understanding the functional impact of genetic variants will advance translational genetics. Examples include deep neural networks trained to discriminate pathogenic from benign variants, such as PrimateAI, SpliceAI, DeepSEA, and DanQ, as well as large language models such as ESM1b that learn the language of proteins to recognize the functional impact of protein changes [45].

Current genetics studies primarily examine the onset of disease. However future studies will focus on disease progression either through collections of specific patient populations, or availability of longitudinal imaging datasets such as that recently announced by UK Biobank. It is very likely that clinical trials will increasingly collect genetic information, particularly if there is a genetic biomarker for patient stratification. However the size of clinical trials is such that power to detect an effect in a single trial is low. More likely, identifying genetic effects on drug efficacy/response will come from large scale analysis of the EHRs in the large biobanks [46].

In addition to implicating targets, genetics can stratify the disease population. In the future we will see many more diseases treated on the basis of single biomarkers or more likely collections of genetic markers derived from polygenic risk analysis [47]. This vision of personalised medicine based on genetic stratification of the disease is already possible in oncology but will become more prevalent elsewhere as molecular signatures of disease are collected. Eventually these signatures may even be derived from machine learning of a combination of genotype with clinical phenotypes and cellular and molecular profiling of the patient and disease tissue. In a future of near universal clinical genome sequencing applied across the health services, genetics can drive changes in clinical care, discovery research, and drug development.
